# Screening and analysis of RNAs associated with activated memory CD4 and CD8 T cells in liver cancer

**DOI:** 10.1186/s12957-021-02461-6

**Published:** 2022-01-04

**Authors:** Zhang Yan, Yin Lijuan, Wu Yinhang, Jin Yin, Xu Jiamin, Wu Wei, Pan Yuefen, Han Shuwen

**Affiliations:** 1 Department of Infectious Disease, Huzhou Central Hospital, Affiliated Central Hospital Huzhou University, No.1558, Sanhuan North Road, Wuxing District, Huzhou, 313000 Zhejiang Province China; 2 Department of Rheumatology, Huzhou Central Hospital, Affiliated Central Hospital Huzhou University, No.1558, Sanhuan North Road, Wuxing District, Huzhou, 313000 Zhejiang Province China; 3 Graduate School of Second Clinical Medicine Faculty, Zhejiang Chinese Medical University, No. 548 Binwen Road, Binjiang District, Hangzhou, 310053 Zhejiang Province China; 4 Department of Laboratory Medicine, Huzhou Hospital of Zhejiang University, Affiliated Central Hospital Huzhou University, No.1558, Sanhuan North Road, Wuxing District, Huzhou, 313000 Zhejiang Province China; 5 Department of Nursing, Shaoxing People’s Hospital, Shaoxing Hospital of Zhejiang University, No. 568 North Zhongxing Road, Yuecheng District, Shaoxing, 312000 Zhejiang Province China; 6 Department of Gastroenterology, Huzhou Hospital of Zhejiang University, Affiliated Central Hospital Huzhou University, No.1558, Sanhuan North Road, Wuxing District, Huzhou, 313000 Zhejiang Province China; 7 Department of Oncology, Huzhou Hospital of Zhejiang University, Affiliated Central Hospital Huzhou University, No.1558, Sanhuan North Road, Wuxing District, Huzhou, 313000 Zhejiang Province China

**Keywords:** Liver cancer, Differentially expressed analysis, Immune-related genes, T cells, Survival analysis, CeRNA network

## Abstract

**Background:**

Liver cancer is one of the most common malignant tumors in the world. T cell-mediated antitumor immune response is the basis of liver cancer immunotherapy.

**Objective:**

To screen and analyze the RNAs associated with activated memory CD4 T cells and CD8 T cells in liver cancer.

**Methods:**

ESTIMATE was used to calculate the stromal and immune scores of tumor samples, which were downloaded from The Cancer Genome Atlas (TCGA). The differentially expressed genes (DEGs) in high and low stromal and immune scores were screened, followed by functional enrichment of overlapped DEGs. We then conducted a survival analysis to identify immune-related prognostic indicators and constructed protein-protein interaction (PPI) networks and ceRNA networks. Finally, chemical small-molecule**–**target interaction pairs associated with liver cancer were screened.

**Results:**

A total of 55,955 stromal-related DEGs and 1811 immune-related DEGs were obtained. The 1238 overlapped DEGs were enriched in 1457 biological process terms and 74 KEGG pathways. In addition, a total of 120 activated memory CD4 T cell-related genes and 309 CD8 T cell-related genes were identified. The survival analysis revealed that upregulated expression of T cell-related genes including EOMES, CST7, and CD5L indicated the favorable prognosis of liver cancer. *EOMES* was regulated by has-miR-23b-3p and has-miR-23b-3p was regulated by lncRNA AC104820.2 in the ceRNA network of activated memory CD4 T cell-related genes. In addition, *EOMES* was regulated by has-miR-23a-3p and has-miR-23a-3p was regulated by lncRNA AC000476.1 in the ceRNA network of CD8 T cells.

**Conclusion:**

T cell-related RNAs EOMES, CST7, CD5L, has-miR-23b-3p, and has-miR-23a-3p may be associated with the prognosis of liver cancer. And the molecular characteristics of these T cell-related genes were plotted.

**Supplementary Information:**

The online version contains supplementary material available at 10.1186/s12957-021-02461-6.

## Abstract (article highlights)

A total of 309 CD8 T cell-related genes and 120 activated memory CD4 T cell-related genes were screened in liver cancer tumor samples.

Forty-four chemical small-molecule–target interaction pairs associated with activated memory CD4 T cells and 276 pairs associated with CD8 T cells were screened.

Upregulated expression of T cell-related genes including EOMES, CST7, and CD5L indicated the favorable prognosis of liver cancer.

**Supplementary Information:**

The online version contains supplementary material available at 10.1186/s12957-021-02461-6.

## Introduction

Liver cancer is one of the ordinary malignant tumors, with high morbidity and mortality [1, 2]. Although the incidence of liver cancer has decreased, there are still approximately 840,000 new cases worldwide every year [3]. Surgical resection and liver transplantation are currently recognized as effective methods for the treatment of liver cancer; however, the 5-year survival rate is only approximately 50%, and the 5-year recurrence rate can reach 60~70% [4]. Epigenetic-related molecules (such as SNRPC) [5], non-coding RNA (such as hsa-mir-221) [6], and immune-related molecules (such as DCK) [7] could be used as potential biomarkers for diagnosis, treatment, and prognosis monitoring in liver cancer. Therefore, it is critically necessary to further explore the molecular pathogenesis of this disease by screening for new molecular biomarkers that could enhance early diagnosis and improve current therapies for liver cancer.

According to the competing endogenous RNA (ceRNA) hypothesis, different RNA transcripts participate in pathological processes, primarily by competing for the binding sites of shared miRNAs. Li et al. have suggested that long noncoding RNA LINC01139 promotes the progression of liver cancer by upregulating *MYBL2* via competitively binding to members of the miR-30 family [8]. In addition, tumor infiltration of immune cells is closely related to the prognosis of tumors and the determination of immunotherapeutic targets [9]. Tumor immune escape is an important characteristic of tumor formation and is related to the decline of the T cell response-ability [10]. T cells are key mediators of tumor destruction and are critical to the specificity of tumor antigen expression [11]. Moreover, numerous studies have shown that the T cell-mediated antitumor immune response is the basis of tumor immunotherapy, which is associated with a favorable prognosis [12]. Parriott et al. found that T cells expressing the chimeric-PD1-Dap10-CD3zeta receptor can reduce the tumor burden in a variety of mouse solid cancer syngeneic models [13]. CD8+ T lymphocytes are essential factors affecting the efficacy of immunotherapy. Donghua et al. identified CD8+ T cell co-expression genes (C1QC, CD3D, GZMA, and PSMB9 ) that promoted infiltration of CD8+ T cells in liver cancer [14]. Immunotherapy is widely used to treat advanced cancer. However, immunotherapy has many limitations, such as low success rate, many complications, and rapid progression. Studies have shown that the low success rate of immunotherapy is related to less T lymphocyte infiltration. Analysis of infiltrating T cell related molecules may be helpful for immunotherapy.

However, there are few reports on the immune-related prognostic indicators of liver cancer. In this study, we aimed to analyze T cells related to tumor immunity and investigate RNAs associated with T cells in liver cancer to identify useful molecular markers related to the prognosis of liver cancer. In the tumor microenvironment, immune cells and stromal cells are two main types of non-tumor components. They are of great significance for the diagnosis and prognosis evaluation. The immune and stroma scores which was based on the ESTIMATE algorithm are the quantification of the immune and stroma components in the tumor microenvironment. The differential genes were divided into upregulated group and downregulated group according to the intermediate value of quantitative scores. In order to find more representative genes, common stroma and immune genes were screened in the upregulated group and downregulated group, respectively. Then, we analyzed the molecular mechanisms associated with T cells in liver cancer based on the representative genes.

## Materials and methods

### Data sources and data preprocessing

The RNA expression RNAseq sequencing data (Counts) and clinical data (phenotype) of LIHC (Version: 07-19-2019) were downloaded from the TCGA database [15] (https://xenabrowser.net/). Fragments Per Kilobase of exon model per Million mapped fragments (FPKM) was used to standardize the original data. The FPKM of a gene in sample was equal to the ratio of the total exon fragments to the reading falling in the genome (map reading (Millions)) and the length of the gene (exon length (KB)). FPKM = Total exon fragments/(Mapped reads×Exon length). The RNA expression RNAseq sequencing data (FPKM) was also downloaded from the TCGA database. A total of 58,387*369 Counts and FPKM representation matrices were obtained. The detection platform of sequencing data was the Illumina HiSeq 2 000 RNA Sequencing platform.

The RNA-Seq was annotated based on the annotation file of the Gencode database [16] (https://www.gencodegenes.org/). Then, genes with “protein_coding” annotations were extracted as mRNA. Meanwhile, RNAs with “TEC,” “known_ncrna,” “macro_lncRNA,” “bidirectional_promoter_lncrna,” and “lncRNA” annotations were extracted as lncRNA, and other genes were defined as “undefined.” Then, the sequencing data log2 (count+1) values of Counts were restored to the count value. Reads and the sequencing data log2 (count+1) values of FPKM were restored to the fpkm value. In the expression profile analysis of the Ensembl_ID, the mapping probe was used to calculate the gene expression value (obtained from the annotation files of the chip platform and microarray dataset) to Symbol_ID. The average value was taken as the level of Ensembl_ID expression when multiple probes matched one Symbol_ID. Lastly, 369 tumor samples, 60,483 ENSEMBLE IDs, and 58,387 RNA symbols were obtained in Table 1. The clinical information related to prognosis in TCGA was also collected in Table 1, containing overall survival (OS) and OS status, and the lifetime was converted from days to months (days/30).

**Table 1:** The basic statistical information of tumor samples in the TCGA-LIHC dataset

TCGA-LIHC	
Tumor	369
ENSEMBLE ID	60,483
RNA symbol	58,387

### Immune score calculation

The stromal and immune scores of all tumor samples were calculated with the Estimation of STromal and Immune cells in MAlignant Tumor tissues using Expression data (ESTIMATE algorithm in R [17].

### Differential expression analysis

The tumor samples were split into high- and low-score groups based on the median value of stromal and immune scores of all tumor samples. The typical Bayesian modified *t* test in limma package [18] (Version 3.40.6) was applied to analyze differentially expressed RNA (DERNA) between high and low immune-score groups with a cutoff of |logFC| > 1 and *P* < 0.05. Lastly, the ggscatter function of the ggpubr package [19] in the R language (Version: 0.2.2) was used to draw a volcano plot of the DERNAs. The DEGs of stromal and immune-score groups were selected as the DEGs related to the immune microenvironment.

### Enrichment analysis

Based on up- and downregulated DEGs, the clusterProfiler package [20] in R was used to perform Gene Ontology (GO) [21] and Kyoto Encyclopedia of Genes and Genomes (KEGG) [22] pathway enrichment analysis with a cutoff of *P* < 0.05 and count ≥ 2.

### Screening of T cell-related genes

RNA-Seq expression profile data were used to target DEGs, and the abundance matrix of the immune cells of the samples was evaluated using the CIBERSORT deconvolution algorithm [23] to analyze the abundance of infiltration of 22 immune cells in tumor samples. The LM22 dataset provided by the Cibersort website was used as the RNA expression signature template following the analysis of the abundance of infiltration of immune cells, and parameters were set as perm = 50 and QN = N. The landscape of the immune cells was then plotted using the ggplot2 package in R. The Pearson correlation coefficient between the expression value of the DEGs and the abundance of immune infiltration in activated memory CD4 T cells and CD8 T cells was calculated, and the activated memory CD4 T and CD8 T cell-related genes were corrected for BH with a threshold value of |r| > 0.3 and *P* < 0.05. Moreover, KEGG pathway analysis on T cell-related genes was carried out based on the carcinoma- and hepatocellular-related pathways in the Comparative Toxicogenomics Database (CTD) [24] (http://ctdbase.org/).

### Survival analysis

Based on the median expression in T cell-related genes, samples were divided into high- and low-expression groups. The survival analysis was then carried out using a Kaplan-Meier plot. Finally, a log-rank statistical test was conducted with a significance threshold of *P* < 0.05.

### PPI network analysis

The STRING database [25] was used to analyze the protein-protein interactions encoded by T cells. The PPI score was set as 0.7 (high-confidence value). Afterward, the PPI network of T cell-related genes was constructed using Cytoscape software [26].

### Prediction of miRNA-target interaction pairs

The miRNAs of the T cell-related genes were predicted using miRWalk 3.0 [27]. Then, the miRNA-target interaction pairs in the TargetScan [28], MiRDB [29], and MirTarBase [30] databases were also obtained using a threshold of score > 0.95. In addition, the HMDD V3.2 database [31] was used to further validate and screen the predicted miRNA with the keywords “Carcinoma, Hepatocellular”.

### Construction of the ceRNA network

The lncRNAs-miRNAs relationship between T cell-related genes was predicted using the DIANA-LncBase database [32]. Finally, the T cell-related gene lncRNA-miRNA-mRNA network was constructed using Cytoscape software.

### Chemical small-molecule–target network analysis

To search for liver cancer-related genes and chemicals, the Comparative Toxicogenomics Database (http://ctd.mdibl.org/) was searched using “Carcinoma, Hepatocellular” as keywords. Genes that were both associated with liver cancer and belonged to the T cell-related genes ceRNA network were used to screen chemical-target pairs. The T cell-related genes chemical small-molecule**–**target network was obtained utilizing the Cytoscape software.

## Results

### The DEGs between the high and low stromal and immune score groups

**Fig. 1:**
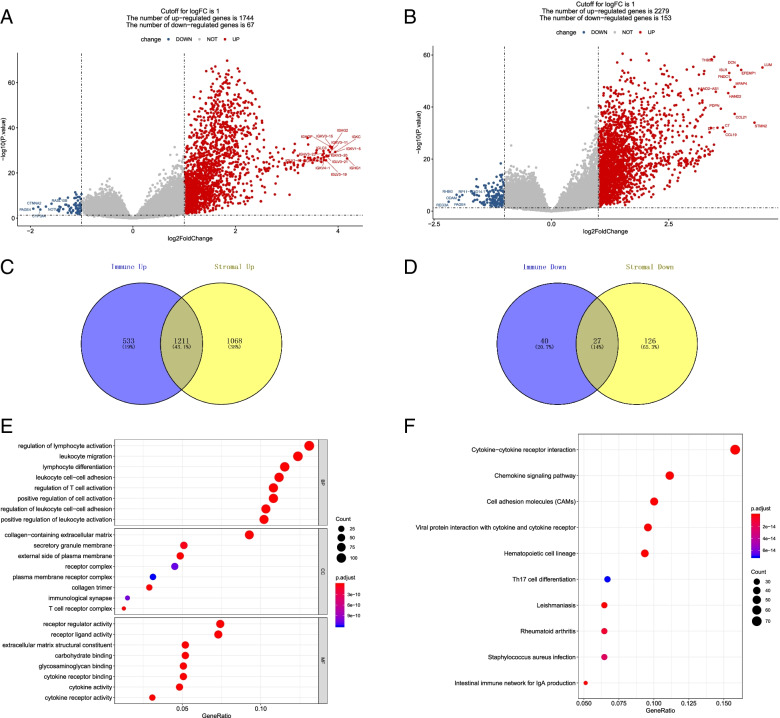
Differentially expressed genes (DEGs) between the high and low stromal and immune score groups. Based on the median value of stromal and immune scores of all tumor samples, the tumor samples were divided into high- and low-score groups. The standard Bayesian modified *t* test in the limma package was applied to analyze differentially expressed RNAs (DERNA) between high and low immune-score groups with a cutoff of |logFC| > 1 and *P* < 0.05. According to the stromal score, there were 55,955 DEGs (2279 upregulated and 153 downregulated) were selected between high and low stromal-score groups. Meanwhile, 1811 DEGs (including 1744 upregulated and 67 downregulated) were selected according to the high and low immune-score groups. In addition, there were 1211 upregulated overlapped DEGs and 27 downregulated overlapped DEGs were selected in the stromal score and immune score groups. In the enrichment analysis of 1238 overlapped DEGs, the GO analysis showed that the 1238 DEGs were mainly enriched in 1457 GO-BPs and 74 KEGG pathways. **A** The DEGs between high and low stromal-score groups. **B** The DEGs between high and low immune-score groups. **C** The upregulated DEGs in the stromal score and immune score groups. D: The downregulated DEGs in the stromal score and immune score groups. **E** The GO analysis of all overlapped DEGs. **F** The KEGG pathways analysis of all overlapped DEGs. Red nodes represent upregulated DEGs and blue nodes represent downregulated DEGs. The size of the ball represents the number of genes enriched in each term. The color of the ball represents the value of the *P* value. DEGs: differentially expressed genes; GO, Gene Ontology; BP, biological process; KEGG, Kyoto Encyclopedia of Genes and Genomes

The aim was to screen immune-related differential genes in liver cancer. In total, 10,221 genes from the TCGA-LIHC dataset were matched by the ESTIMATE algorithm, and the number of unmatched genes was 191. There were 1811 DEGs (of which 1744 and 67 were up- and downregulated, respectively) were selected according to the high and low immune score groups (Fig. 1A and Supplementary file 1). Meanwhile, a total of 55,955 DEGs (of which 2279 and 153 were up- and downregulated, respectively) were selected between high and low stromal score groups (Fig. 1B and Supplementary file 2). In addition, 1211 overlapped upregulated DEGs and 27 overlapped downregulated DEGs were selected in the stromal score and immune score groups (Fig. 1C, D). The names of DEGs are shown in Supplementary file 3. In the enrichment analysis of 1238 overlapped DEGs, the GO analysis showed that the 1238 DEGs were mainly enriched in 1457 GO-biological processes (BPs; e.g., regulation of lymphocyte activation, leukocyte migration, and lymphocyte differentiation) and 74 KEGG pathways [e.g., cell-adhesion molecules (CAMs)] (Fig. 1E, F).

### Identification of T cell-related genes

**Fig. 2:**
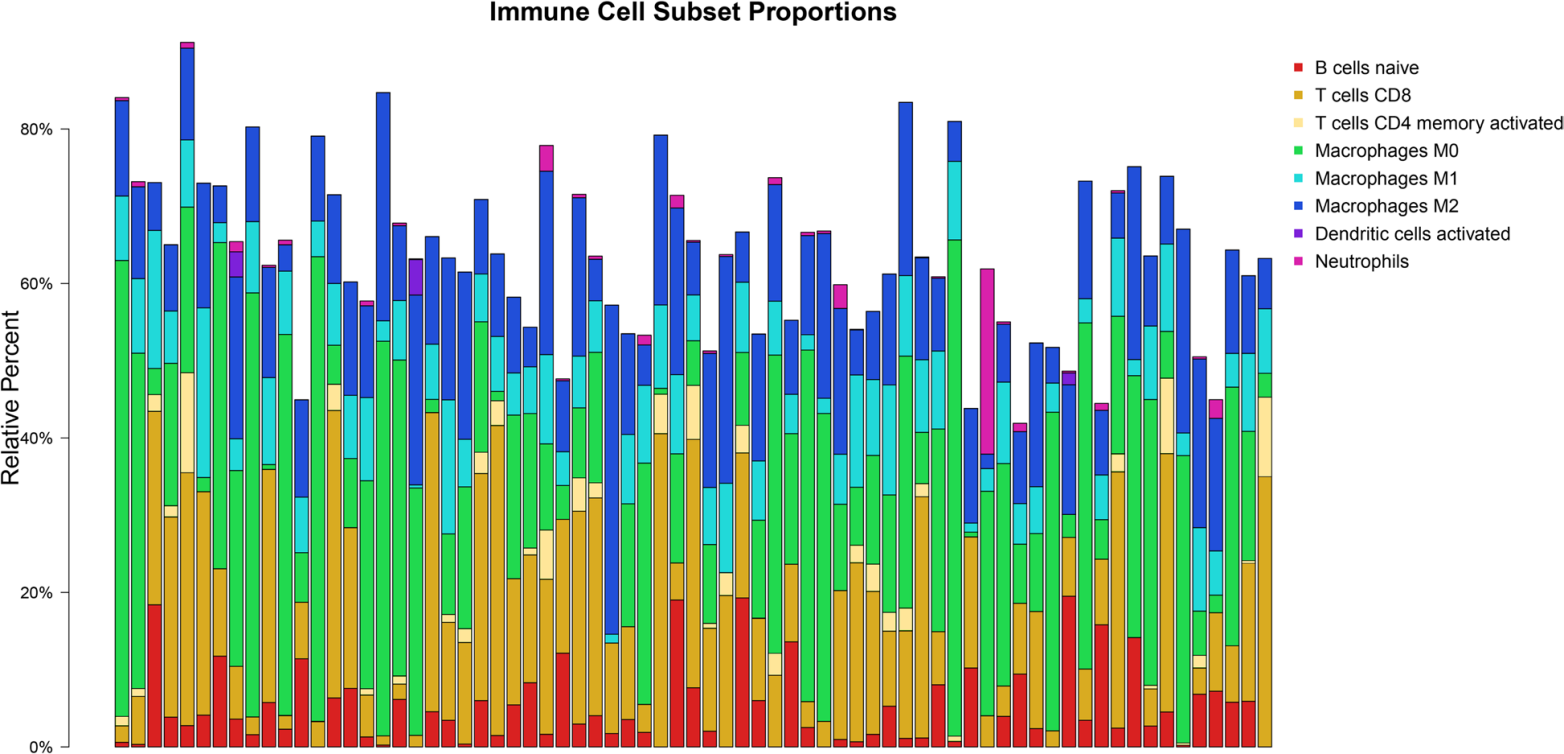
The landscape of the immune cells. To analyze the abundance of infiltration of the immune cells in the samples, RNA-Seq expression profile data were used to target DEGs, and the abundance matrix of the immune cells was evaluated through the CIBERSORT deconvolution algorithm. Finally, the abundance of infiltration of immune cells (including naïve B cells, memory B cells, CD8 T cells, activated memory CD4T cells, M0 macrophages, M1 macrophages, M2 macrophages, activated dendritic cells, and neutrophils) was determined

**Fig. 3:**
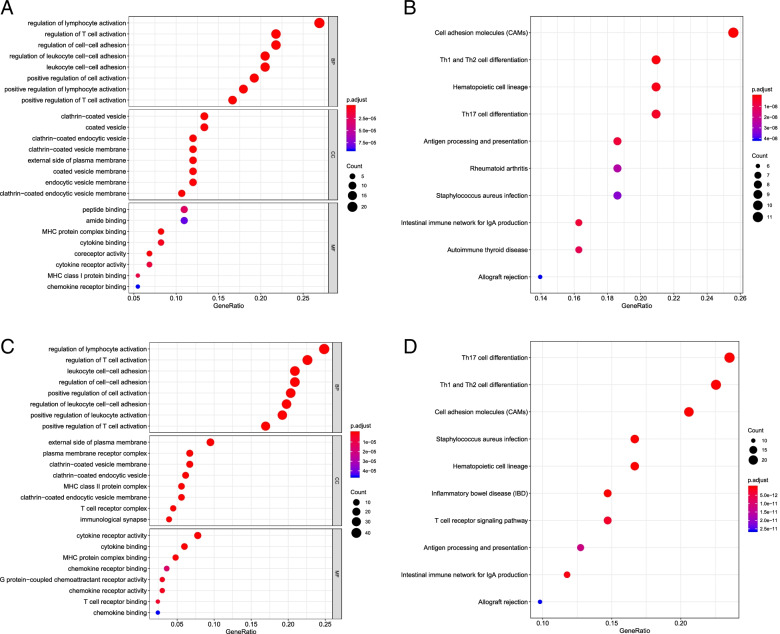
Enrichment analysis of activated memory CD4 T cells and CD8 T cell-related genes. The clusterProfiler package in R was used to perform GO and KEGG pathway enrichment analysis with a cutoff of *P* < 0.05 and count ≥ 2. In the enrichment analysis of activated memory CD4 T cells and CD8 T cell-related genes, activated memory CD4 T cell-related genes were enriched in 406 GO-BPs (e.g., regulation of lymphocyte activation, regulation of T cell activation, and regulation of cell-cell adhesion) and 35 KEGG pathways [e.g., CAMs, Th1 and Th2 cell differentiation and Hematopoietic cell lineage]. In addition, CD8 T cell-related genes were involved in 596 GO-BPs (e.g., regulation of lymphocyte activation, regulation of T cell activation, and regulation of cell-cell adhesion) and 42 KEGG pathways [e.g., Th17 cell differentiation, Th1 and Th2 cell differentiation, and CAMs]. **A** The GO analysis of activated memory CD4 T cell-related genes. **B** The GO analysis of CD8 T cell-related genes. **C** The KEGG pathways analysis activated memory CD4 T cell-related genes. **D** The KEGG pathways analysis CD8 T cell-related genes. The size of the ball represents the number of genes enriched in each term. The color of the ball represents the value of the *P* value. GO, Gene Ontology; BP, biological process; KEGG, Kyoto Encyclopedia of Genes and Genomes

T cells played an important role in liver cancer occurrence and development. The aim was to further analyze T cell-related genes in liver cancer. The abundance of immune-cell infiltration in tumor samples was estimated and the immune-cell landscape was shown in Fig. 2. Meanwhile, according to the Pearson correlation coefficient, a total of 120 activated memory CD4 T cell-related genes (Supplementary file 4) and 309 CD8 T cell-related genes were identified (Supplementary file 5). In the enrichment analysis of activated memory CD4 T cells and CD8 T cell-related genes, activated memory CD4 T cell-related genes were enriched in 406 GO-BPs and 35 KEGG pathways [e.g., CAMs, Th1 and Th2 cell differentiation, and Hematopoietic cell lineage], the Top 8 GO terms and Top 10 KEGG pathways were shown in Fig. 3A and B, respectively. In addition, CD8 T cell-related genes were involved in 596 GO-BPs and 42 KEGG pathways [e.g., Th17 cell differentiation, Th1 and Th2 cell differentiation, and CAMs], the Top 8 GO terms and Top 10 KEGG pathways were shown in Fig. 3C and D, respectively. In addition, based on the CTD database, activated memory CD4 T cell-related genes were involved in 31 liver cancer-related pathways [e.g., CAMs, Th1 and Th2 cell differentiation, hematopoietic cell lineage] (Table 2), and CD8 T cell-related genes were involved in 36 liver cancer-related pathways [e.g., Th17 cell differentiation, Th1 and Th2 cell differentiation, and CAMs] (Table 3).

**Table 2:** The KEGG pathway analysis of activated memory CD4 T cells and CD8 T cell-related genes in the Comparative Toxicogenomics Database. The KEGG pathway analysis of activated memory CD4 T cell-related genes

ID	Description	*p* value	*p*.adjust	*q* value	Count	gene_symbol
hsa04514	Cell adhesion molecules (CAMs)	1.98E-10	1.98E-10	8.55E-09	11	CTLA4/CD8A/CD8B/TIGIT/HLA-DRB1/ICOS/CD86/HLA-DPB1/HLA-DQA1/HLA-DRA/HLA-DPA1
hsa04658	Th1 and Th2 cell differentiation	9.90E-10	9.90E-10	2.14E-08	9	IL12RB1/TBX21/HLA-DRB1/CD3D/HLA-DPB1/HLA-DQA1/HLA-DRA/HLA-DPA1/LCK
hsa04640	Hematopoietic cell lineage	1.92E-09	1.92E-09	2.76E-08	9	CD8A/CD8B/HLA-DRB1/CD3D/HLA-DPB1/HLA-DQA1/HLA-DRA/FCGR1A/HLA-DPA1
hsa04659	Th17 cell differentiation	3.86E-09	3.86E-09	4.16E-08	9	IL12RB1/TBX21/HLA-DRB1/CD3D/HLA-DPB1/HLA-DQA1/HLA-DRA/HLA-DPA1/LCK
hsa04060	Cytokine-cytokine receptor interaction	2.07E-05	2.07E-05	3.58E-05	9	CXCL9/CCL4/CCL5/IL12RB1/CD27/CXCR6/CXCL13/CXCR3/CCR5
hsa04612	Antigen processing and presentation	6.37E-09	6.37E-09	4.58E-08	8	CD8A/CD8B/CD74/HLA-DRB1/HLA-DPB1/HLA-DQA1/HLA-DRA/HLA-DPA1
hsa05323	Rheumatoid arthritis	2.61E-08	2.61E-08	1.41E-07	8	CTLA4/CCL5/HLA-DRB1/CD86/HLA-DPB1/HLA-DQA1/HLA-DRA/HLA-DPA1
hsa05322	Systemic lupus erythematosus	4.29E-07	4.29E-07	1.23E-06	8	HLA-DRB1/CD86/HLA-DPB1/FCGR3A/HLA-DQA1/HLA-DRA/FCGR1A/HLA-DPA1
hsa04145	Phagosome	1.19E-06	1.19E-06	2.70E-06	8	HLA-DRB1/HLA-DPB1/FCGR3A/HLA-DQA1/OLR1/HLA-DRA/FCGR1A/HLA-DPA1
hsa05152	Tuberculosis	4.24E-06	4.24E-06	8.72E-06	8	CD74/HLA-DRB1/HLA-DPB1/FCGR3A/HLA-DQA1/HLA-DRA/FCGR1A/HLA-DPA1
hsa04062	Chemokine signaling pathway	6.10E-06	6.10E-06	1.14E-05	8	CXCL9/CCL4/CCL5/CXCR6/CXCL13/CXCR3/CCR5/GNGT2
hsa04672	Intestinal immune network for IgA production	5.76E-09	5.76E-09	4.58E-08	7	HLA-DRB1/ICOS/CD86/HLA-DPB1/HLA-DQA1/HLA-DRA/HLA-DPA1
hsa05320	Autoimmune thyroid disease	1.02E-08	1.02E-08	6.27E-08	7	CTLA4/HLA-DRB1/CD86/HLA-DPB1/HLA-DQA1/HLA-DRA/HLA-DPA1
hsa05321	Inflammatory bowel disease (IBD)	4.38E-08	4.38E-08	1.72E-07	7	IL12RB1/TBX21/HLA-DRB1/HLA-DPB1/HLA-DQA1/HLA-DRA/HLA-DPA1
hsa05140	Leishmaniasis	1.44E-07	1.44E-07	4.45E-07	7	HLA-DRB1/HLA-DPB1/FCGR3A/HLA-DQA1/HLA-DRA/FCGR1A/HLA-DPA1
hsa04660	T cell receptor signaling pathway	1.14E-06	1.14E-06	2.70E-06	7	CTLA4/CD8A/CD8B/ICOS/CD3D/LCP2/LCK
hsa04380	Osteoclast differentiation	4.63E-06	4.63E-06	9.08E-06	7	SIRPG/LILRB2/LILRB1/FCGR3A/FCGR1A/LCP2/LCK
hsa05166	Human T cell leukemia virus 1 infection	0.000148	0.000148	0.000237	7	HLA-DRB1/CD3D/HLA-DPB1/HLA-DQA1/HLA-DRA/HLA-DPA1/LCK
hsa05168	Herpes simplex virus 1 infection	0.014966	0.014966	0.018997	7	CCL5/CD74/HLA-DRB1/HLA-DPB1/HLA-DQA1/HLA-DRA/HLA-DPA1
hsa05330	Allograft rejection	4.14E-08	4.14E-08	1.72E-07	6	HLA-DRB1/CD86/HLA-DPB1/HLA-DQA1/HLA-DRA/HLA-DPA1
hsa05332	Graft-versus-host disease	6.67E-08	6.67E-08	2.40E-07	6	HLA-DRB1/CD86/HLA-DPB1/HLA-DQA1/HLA-DRA/HLA-DPA1
hsa04940	Type I diabetes mellitus	8.97E-08	8.97E-08	2.98E-07	6	HLA-DRB1/CD86/HLA-DPB1/HLA-DQA1/HLA-DRA/HLA-DPA1
hsa05416	Viral myocarditis	6.88E-07	6.88E-07	1.75E-06	6	HLA-DRB1/CD86/HLA-DPB1/HLA-DQA1/HLA-DRA/HLA-DPA1
hsa05145	Toxoplasmosis	2.67E-05	2.67E-05	4.44E-05	6	HLA-DRB1/HLA-DPB1/HLA-DQA1/HLA-DRA/CCR5/HLA-DPA1
hsa05164	Influenza A	0.000272	0.000272	0.000419	6	CCL5/HLA-DRB1/HLA-DPB1/HLA-DQA1/HLA-DRA/HLA-DPA1
hsa05169	Epstein-Barr virus infection	0.000665	0.000665	0.000989	6	HLA-DRB1/CD3D/HLA-DPB1/HLA-DQA1/HLA-DRA/HLA-DPA1
hsa05310	Asthma	5.48E-07	5.48E-07	1.48E-06	5	HLA-DRB1/HLA-DPB1/HLA-DQA1/HLA-DRA/HLA-DPA1
hsa04620	Toll-like receptor signaling pathway	0.002276	0.002276	0.003274	4	CXCL9/CCL4/CCL5/CD86
hsa04650	Natural killer cell mediated cytotoxicity	0.005222	0.005222	0.007042	4	FCGR3A/SH2D1A/LCP2/LCK
hsa04623	Cytosolic DNA-sensing pathway	0.004625	0.004625	0.006439	3	CCL4/CCL5/ZBP1
hsa04662	B cell receptor signaling pathway	0.00961	0.00961	0.012568	3	LILRB2/CD72/LILRB1

**Table 3:** The KEGG pathway analysis of activated memory CD4 T cells and CD8 T cell-related genes in the Comparative Toxicogenomics Database. The KEGG pathway analysis of CD8 T cell-related genes

Description	*p* value	*p*.adjust	*q* value	gene_symbol
Th17 cell differentiation	4.53E-24	4.53E-24	2.55E-22	TBX21/IL12RB1/ZAP70/CD3D/LCK/HLA-DPB1/HLA-DQB1/HLA-DRA/HLA-DPA1/HLA-DRB1/CD3E/IL21R/HLA-DRB5/JAK3/HLA-DMB/IL2RB/HLA-DOA/CD247/HLA-DQA1/CD3G/IL2RG/GATA3/IRF4/IL2RA
Th1 and Th2 cell differentiation	2.90E-24	2.90E-24	2.55E-22	TBX21/IL12RB1/ZAP70/CD3D/LCK/HLA-DPB1/HLA-DQB1/HLA-DRA/HLA-DPA1/HLA-DRB1/RUNX3/CD3E/HLA-DRB5/JAK3/HLA-DMB/IL2RB/HLA-DOA/CD247/HLA-DQA1/CD3G/IL2RG/GATA3/IL2RA
Cell adhesion molecules (CAMs)	8.08E-17	8.08E-17	3.03E-15	CD8B/CD8A/CTLA4/TIGIT/ICOS/PDCD1/HLA-DPB1/HLA-DQB1/HLA-DRA/HLA-DPA1/HLA-DRB1/CD2/HLA-DRB5/HLA-DMB/HLA-DOA/CD6/ICAM3/HLA-DQA1/CD226/CD86/SELPLG
Cytokine-cytokine receptor interaction	6.07E-10	6.07E-10	3.42E-09	CCL4/CXCL9/CCL5/CD27/IL12RB1/CXCR3/CXCR6/CCR5/TNFRSF8/IL21R/IL2RB/CXCL13/IL10RA/CCR2/IL18RAP/CXCL11/TNFRSF17/IL2RG/IL16/IL2RA
Human T cell leukemia virus 1 infection	2.30E-10	2.30E-10	1.44E-09	CD3D/LCK/HLA-DPB1/HLA-DQB1/HLA-DRA/HLA-DPA1/HLA-DRB1/PIK3CD/CD3E/HLA-DRB5/JAK3/HLA-DMB/IL2RB/HLA-DOA/HLA-DQA1/CD3G/IL2RG/IL2RA
Hematopoietic cell lineage	3.87E-15	3.87E-15	7.27E-14	CD8B/CD8A/CD3D/HLA-DPB1/HLA-DQB1/HLA-DRA/HLA-DPA1/HLA-DRB1/CD2/CD3E/HLA-DRB5/HLA-DMB/HLA-DOA/HLA-DQA1/FCGR1A/CD3G/IL2RA
Epstein-Barr virus infection	4.09E-09	4.09E-09	2.00E-08	CD3D/HLA-DPB1/HLA-DQB1/HLA-DRA/HLA-DPA1/HLA-DRB1/RUNX3/PIK3CD/CD3E/HLA-DRB5/JAK3/HLA-DMB/HLA-DOA/CD247/HLA-DQA1/CD3G
Inflammatory bowel disease (IBD)	1.70E-15	1.70E-15	4.77E-14	TBX21/IL12RB1/HLA-DPB1/HLA-DQB1/HLA-DRA/HLA-DPA1/HLA-DRB1/IL21R/HLA-DRB5/HLA-DMB/HLA-DOA/HLA-DQA1/IL18RAP/IL2RG/GATA3
T cell receptor signaling pathway	2.60E-12	2.60E-12	3.67E-11	CD8B/CD8A/CTLA4/ZAP70/ICOS/PDCD1/CD3D/LCK/PIK3CD/CD3E/LCP2/ITK/CD247/VAV1/CD3G
Systemic lupus erythematosus	9.81E-11	9.81E-11	7.17E-10	HLA-DPB1/HLA-DQB1/HLA-DRA/HLA-DPA1/HLA-DRB1/C1QB/C1QA/C1QC/HLA-DRB5/FCGR3A/HLA-DMB/HLA-DOA/HLA-DQA1/FCGR1A/CD86
Tuberculosis	7.02E-09	7.02E-09	3.29E-08	CD74/HLA-DPB1/HLA-DQB1/HLA-DRA/HLA-DPA1/HLA-DRB1/CIITA/HLA-DRB5/FCGR3A/HLA-DMB/CORO1A/HLA-DOA/IL10RA/HLA-DQA1/FCGR1A
Chemokine signaling pathway	1.37E-08	1.37E-08	6.16E-08	CCL4/CXCL9/CCL5/CXCR3/CXCR6/CCR5/PIK3CD/JAK3/GNGT2/CXCL13/ITK/VAV1/CCR2/CXCL11/DOCK2
Antigen processing and presentation	1.24E-11	1.24E-11	1.55E-10	CD8B/CD8A/CD74/HLA-DPB1/HLA-DQB1/HLA-DRA/HLA-DPA1/HLA-DRB1/CIITA/HLA-DRB5/HLA-DMB/HLA-DOA/HLA-DQA1
Phagosome	5.66E-08	5.66E-08	2.36E-07	HLA-DPB1/HLA-DQB1/HLA-DRA/HLA-DPA1/HLA-DRB1/HLA-DRB5/FCGR3A/HLA-DMB/CORO1A/HLA-DOA/HLA-DQA1/FCGR1A/NCF2
Herpes simplex virus 1 infection	0.00945	0.00945	0.02596	CCL5/CD74/HLA-DPB1/HLA-DQB1/HLA-DRA/HLA-DPA1/HLA-DRB1/PIK3CD/HLA-DRB5/HLA-DMB/HLA-DOA/HLA-DQA1/POU2F2
Intestinal immune network for IgA production	6.14E-13	6.14E-13	9.88E-12	ICOS/HLA-DPB1/HLA-DQB1/HLA-DRA/HLA-DPA1/HLA-DRB1/HLA-DRB5/HLA-DMB/HLA-DOA/HLA-DQA1/CD86/TNFRSF17
Leishmaniasis	1.82E-10	1.82E-10	1.21E-09	HLA-DPB1/HLA-DQB1/HLA-DRA/HLA-DPA1/HLA-DRB1/HLA-DRB5/FCGR3A/HLA-DMB/HLA-DOA/HLA-DQA1/FCGR1A/NCF2
Rheumatoid arthritis	1.75E-09	1.75E-09	9.36E-09	CTLA4/CCL5/HLA-DPB1/HLA-DQB1/HLA-DRA/HLA-DPA1/HLA-DRB1/HLA-DRB5/HLA-DMB/HLA-DOA/HLA-DQA1/CD86
Toxoplasmosis	1.52E-08	1.52E-08	6.56E-08	HLA-DPB1/HLA-DQB1/HLA-DRA/HLA-DPA1/HLA-DRB1/CIITA/CCR5/HLA-DRB5/HLA-DMB/HLA-DOA/IL10RA/HLA-DQA1
Influenza A	1.52E-06	1.52E-06	5.91E-06	CCL5/HLA-DPB1/HLA-DQB1/HLA-DRA/HLA-DPA1/HLA-DRB1/CIITA/PIK3CD/HLA-DRB5/HLA-DMB/HLA-DOA/HLA-DQA1
Autoimmune thyroid disease	4.21E-11	4.21E-11	3.95E-10	CTLA4/HLA-DPB1/HLA-DQB1/HLA-DRA/HLA-DPA1/HLA-DRB1/HLA-DRB5/HLA-DMB/HLA-DOA/HLA-DQA1/CD86
Osteoclast differentiation	6.09E-07	6.09E-07	2.45E-06	SIRPG/LILRB2/LCK/LILRB1/PIK3CD/FCGR3A/LCP2/FCGR1A/LILRA1/LILRA5/NCF2
Allograft rejection	2.62E-11	2.62E-11	2.68E-10	HLA-DPB1/HLA-DQB1/HLA-DRA/HLA-DPA1/HLA-DRB1/HLA-DRB5/HLA-DMB/HLA-DOA/HLA-DQA1/CD86
Graft-versus-host disease	6.01E-11	6.01E-11	5.21E-10	HLA-DPB1/HLA-DQB1/HLA-DRA/HLA-DPA1/HLA-DRB1/HLA-DRB5/HLA-DMB/HLA-DOA/HLA-DQA1/CD86
Type I diabetes mellitus	1.01E-10	1.01E-10	7.17E-10	HLA-DPB1/HLA-DQB1/HLA-DRA/HLA-DPA1/HLA-DRB1/HLA-DRB5/HLA-DMB/HLA-DOA/HLA-DQA1/CD86
Viral myocarditis	3.31E-09	3.31E-09	1.69E-08	HLA-DPB1/HLA-DQB1/HLA-DRA/HLA-DPA1/HLA-DRB1/HLA-DRB5/HLA-DMB/HLA-DOA/HLA-DQA1/CD86
Asthma	1.02E-10	1.02E-10	7.17E-10	HLA-DPB1/HLA-DQB1/HLA-DRA/HLA-DPA1/HLA-DRB1/HLA-DRB5/HLA-DMB/HLA-DOA/HLA-DQA1
Chagas disease (American trypanosomiasis)	5.47E-06	5.47E-06	2.05E-05	CCL5/CD3D/C1QB/PIK3CD/C1QA/CD3E/C1QC/CD247/CD3G
Natural killer cell mediated cytotoxicity	0.000256	0.000256	0.000875	ZAP70/SH2D1A/LCK/PIK3CD/FCGR3A/LCP2/CD247/VAV1
Measles	0.000366	0.000366	0.001211	CD3D/PIK3CD/CD3E/JAK3/IL2RB/CD3G/IL2RG/IL2RA
JAK-STAT signaling pathway	0.001058	0.001058	0.003309	IL12RB1/PIK3CD/IL21R/JAK3/IL2RB/IL10RA/IL2RG/IL2RA
B cell receptor signaling pathway	7.87E-05	7.87E-05	0.000277	CD72/LILRB2/LILRB1/PIK3CD/VAV1/LILRA1/LILRA5
Toll-like receptor signaling pathway	0.002081	0.002081	0.006167	CCL4/CXCL9/CCL5/PIK3CD/CD86/CXCL11
Fc gamma R-mediated phagocytosis	0.006618	0.006618	0.019112	PIK3CD/FCGR3A/VAV1/FCGR1A/DOCK2
Prion diseases	0.000973	0.000973	0.00313	CCL5/C1QB/C1QA/C1QC
Cytosolic DNA-sensing pathway	0.008425	0.008425	0.023723	CCL4/CCL5/ZBP1/AIM2

### T cells associated with prognosis of liver cancer

Liver cancer was an often fatal malignant with poor prognosis. The purpose was to further analyze the role of T cell-related genes in the prognosis of hepatocellular carcinoma. A total of 363 sets of survival information from patients with liver cancer was obtained. Moreover, nine activated memory CD4 T cell-related genes were associated with liver cancer prognosis [e.g., eomesodermin (*EOMES*), glutathione S-transferase (*CST7*), and adhesion G protein-coupled receptor E2 (*EMR2*)] (Table 4). In addition, there were 30 CD8 T cell-related genes associated with liver cancer prognosis [e.g., CD5 molecules like *CD5L*, *EOMES*, and *CST7*] (Table 5). Upregulated expression of T cell-related genes including EOMES, CST7, and CD5L indicated the favorable prognosis of liver cancer. Downregulated expression of T cell-related genes including NCF2 and HTAR3 indicated the poor prognosis of liver cancer.

**Table 4:** Activated memory CD4 T cells and CD8 T cells associated with prognosis of liver cancer. Activated memory CD4 T cell-related genes associated with liver cancer prognosis

Names	*p*	High.median	Low.median
EOMES	0.002087	82	41
CST7	0.008028	71	41
EMR2	0.013856	48	83
TRGC2	0.018419	71	47
IGLV7-43	0.021593	72	47
GPR171	0.02511	71	52
TRBV9	0.030513	82	55
ANKRD22	0.03693	46	85
PYHIN1	0.042679	82	52

**Table 5:** Activated memory CD4 T cells and CD8 T cells associated with prognosis of liver cancer. CD8 T cell-related genes associated with liver cancer prognosis

Names	*p*	High.median	Low.median
CD5L	0.001343	82	41
EOMES	0.002087	82	41
IL18RAP	0.002115	71	38
TRBV25-1	0.005602	82	41
NCF2	0.006911	47	85
CST7	0.008028	71	41
ZAP70	0.012036	82	47
IGHV3-7	0.012411	82	47
TRAV19	0.01277	72	41
IGLV3-19	0.016579	82	47
TRGC2	0.018419	71	47
HTRA3	0.018774	47	72
C16orf54	0.019534	71	47
IGKV2D-29	0.01972	71	52
IGLV7-43	0.021593	72	47
ICAM3	0.024014	71	52
RP11-367G6.3	0.024711	62	41
GPR171	0.02511	71	52
RP11-1094M14.8	0.025113	71	52
S1PR4	0.026595	62	52
PTGDR	0.029741	62	47
TRBV9	0.030513	82	55
SLAMF6	0.030972	82	52
GZMK	0.033125	82	47
UBASH3A	0.033482	82	47
TRGC1	0.034744	71	41
TRAV1-2	0.037557	71	41
PYHIN1	0.042679	82	52
THEMIS	0.045249	82	47
CD69	0.046828	71	52

### PPI network of the T cell-related genes

**Fig. 4:**
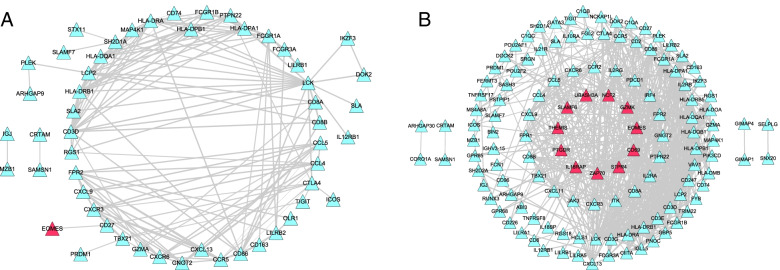
The PPI network of activated memory CD4 and CD8 T cell-related genes. The STRING database was used to analyze protein-protein interactions encoded by activated memory CD4 T cells and CD8 T cells. The PPI score was set as 0.7 (high-confidence value). Afterward, the PPI networks of activated memory CD4 T cells and CD8 T cell-related genes were constructed using Cytoscape software. PPI network analysis of activated memory CD4 T cell-related genes revealed 53 nodes and 162 interaction pairs. The CD8 T cell-related gene PPI network contained 127 nodes and 613 interaction pairs. **A** The PPI network of activated memory CD4 T cell-related genes. **B** The PPI network of CD8 T cell-related genes. Red nodes represent survival-related DEGs, triangles represent upregulated DEGs, and blue nodes represent other DEGs. The size of nodes represents the value. Larger nodes indicate a larger value. PPI, protein-protein interaction; STRING, Search Tool for the Retrieval of Interacting Genes

The PPI network was constructed to plot the characteristics of these molecules. PPI network analysis of activated memory CD4 T cell-related genes revealed 53 nodes, including one survival-related gene (*EOMES*), and 162 interaction pairs (Fig. 4A). The CD8 T cell-related gene PPI network contained 127 nodes, including 11 survival-related genes [e.g., *EOMES*, CD69 molecule (*CD69*), and zeta chain of T cell receptor-associated protein kinase 70 (*ZAP70*)], and 613 interaction pairs (Fig. 4B).

### CeRNA network of the T cell-related genes

**Fig. 5:**
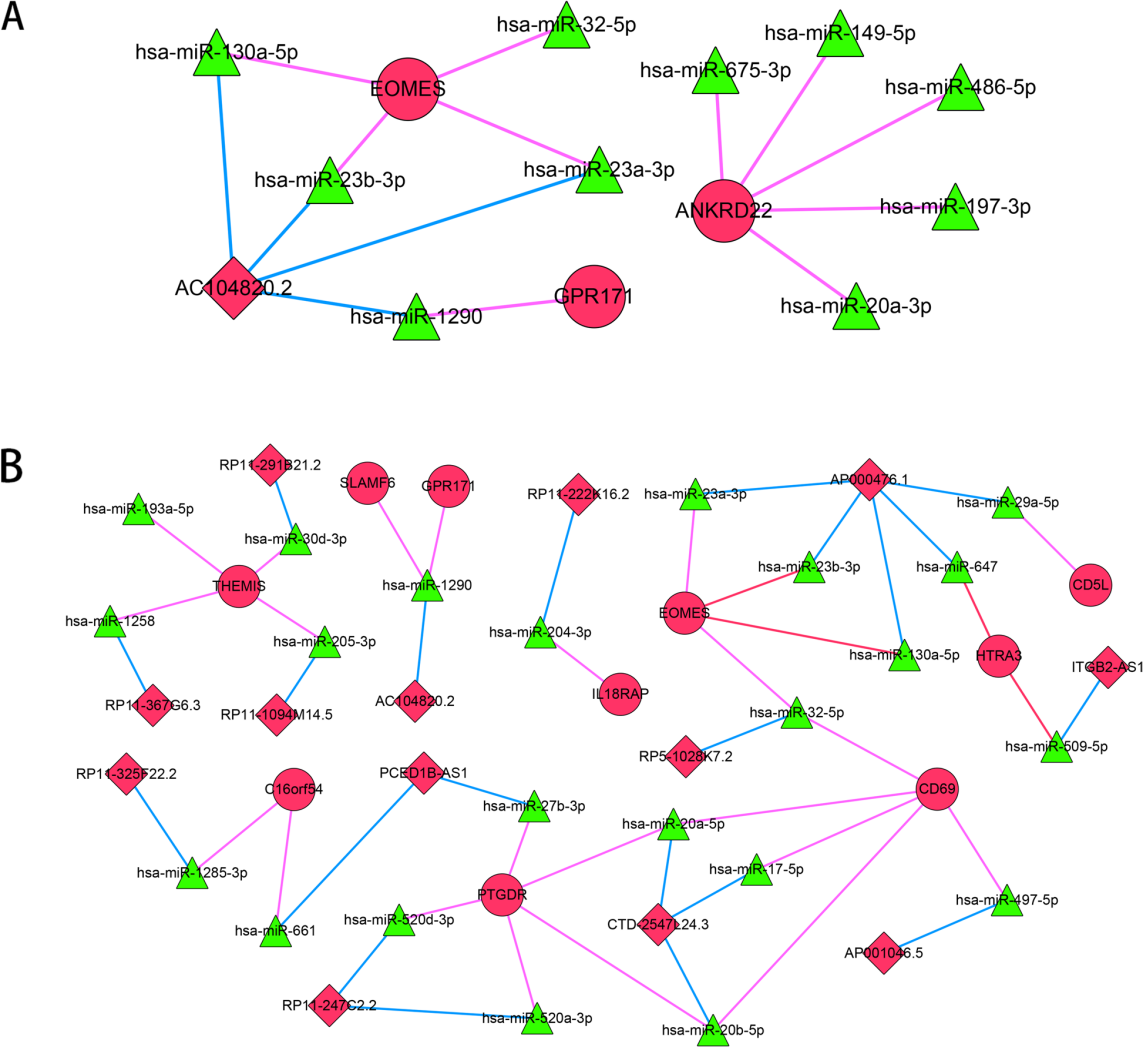
CeRNA network of activated memory CD4 T cells and CD8 T cell-related genes. The miRNAs of activated memory CD4 T cells and CD8 T cell-related genes were predicted using miRWalk 3.0, and miRNA-target interaction pairs in the TargetScan, MiRDB, and MirTarBase databases were obtained using a threshold of score > 0.95. In addition, the HMDD V3.2 database was used to further validate and screen the predicted miRNA using the keywords “Carcinoma, Hepatocellular”. The lncRNAs-miRNAs relationship between activated memory CD4 T cells and CD8 T cell-related genes was predicted using the DIANA-LncBase database. Finally, activated memory CD4 T cell- and CD8 T cell-related gene lncRNA-miRNA-mRNA network was constructed utilizing Cytoscape software. *EOMES* was regulated by has-miR-23b-3p and has-miR-23b-3p were regulated by lncRNA AC104820.2. *EOMES* was regulated by has-miR-23a-3p and has-miR-23a-3p was regulated by lncRNA AC000476.1. **A** ceRNA network of activated memory CD4 T cell-related genes. **B** ceRNA network of CD8 T cell-related genes. Red nodes represent upregulated DEGs, green triangles represent miRNA, and the red rhombi represent upregulated lncRNAs

The ceRNA network was constructed to describe the regulatory effect of non-coding RNA on T cell-related differential molecules. Based on the HMDD database, ten miRNA-mRNA relationships of activated memory CD4 T cell-related genes were obtained (10 miRNAs and three target genes). Also, 26 miRNA-mRNA relationships of CD8 T cell-related genes were obtained (22 miRNAs and 10 target genes). Four miRNA-lncRNA relationships of activated memory CD4 T cell-related genes were obtained (four miRNAs and one lncRNA). Furthermore, 21 miRNA-lncRNA relationships of CD8 T cell-related genes were obtained (21 miRNAs and 13 lncRNAs). Based on the ten miRNA-mRNA relationships and four miRNA-lncRNA relationships of activated memory CD4 T cell-related genes, a ceRNA network of activated memory CD4 T cell-related genes was constructed (Fig. 5A). Here, *EOMES* was regulated by has-miR-23b-3p and has-miR-23b-3p was regulated by lncRNA AC104820.2. In addition, the ceRNA network of CD8 T cell-related genes was constructed among 26 miRNA-mRNA relationships and 21 miRNA-lncRNA relationships (Fig. 5B). Here, *EOMES* was regulated by has-miR-23a-3p and has-miR-23a-3p was regulated by lncRNA AC000476.1.

### Chemical small-molecule–target network analysis of T cell-related genes

**Fig. 6:**
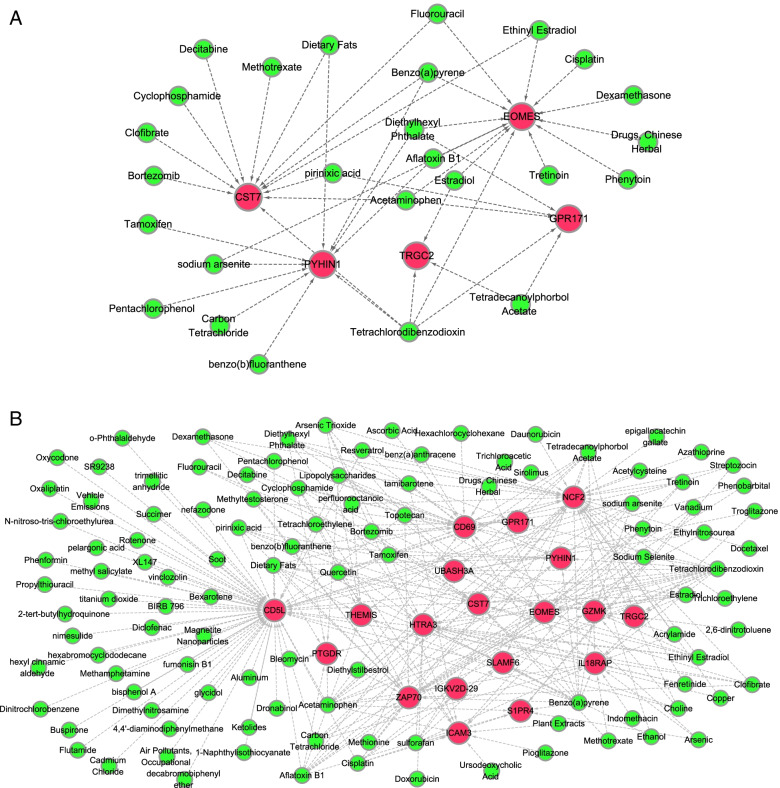
Chemical small-molecule–target network analysis of activated memory CD4 T cells and CD8 T cell-related genes. To search for liver cancer-related genes and chemicals, the Comparative Toxicogenomics Database was searched using “Carcinoma, Hepatocellular” as keywords. Genes that were both associated with liver cancer, and belonged to the T cell-related genes ceRNA network, were used to screen chemical-target pairs. The T cell-related genes chemical small-molecule**–**target network was obtained utilizing the Cytoscape software. There were 44 chemical small-molecule**–**target interaction pairs associated with activated memory CD4 T cells, including five mRNAs and 26 chemical small molecules. In addition, there were 276 CD8 T cell-associated chemical small-molecule**–**target interaction pairs, containing 19 mRNAs and 110 chemical small molecules. **A** The chemical small-molecule**–**target network of genes in activated memory CD4 T cells. **B** The chemical small-molecule**–**target network of genes in CD8 T cells. Red nodes represent survival-related upregulated DEGs, and green nodes represent chemical small molecules. The size of nodes represents the value, such that larger nodes indicate a larger value

The chemical small-molecule–target network was constructed to find agents that regulate these differentially expressed genes. There were 44 chemical small-molecule**–**target interaction pairs associated with activated memory CD4 T cells (Fig. 6A), including five mRNAs and 26 chemical small molecules. In addition, there were 276 CD8 T cell-associated chemical small-molecule**–**target interaction pairs, containing 19 mRNAs and 110 chemical small molecules (Fig. 6B).

## Discussion

In this study, a total of 55,955 stromal-related DEGs and 1811 immune-related DEGs were obtained. Then, the 1238 overlapped DEGs were enriched in 1457 BPs and 74 KEGG pathways. In addition, a total of 120 activated memory CD4 T cell-related genes and 309 CD8 T cell-related genes were identified. The survival analysis showed that the T cell-related genes *EOMES*, *CST7*, *CD5L*, and *EMR2* were associated with the prognosis of liver cancer. Our analysis recommended the AC104820.2-has-miR-23b-3p-*EOMES* axis and the AC000476.1-has-miR-23a-3p-*EOMES* axis from activated memory CD4 T cell- and CD8 T cell-related ceRNAs, respectively.

A total of 120 activated memory CD4 T cell-related genes and 309 CD8 T cell-related genes were identified in this study. Moreover, T cell-related genes were involved in Th1 and Th2 cell differentiation and CAM signaling pathways. Wang et al. found that abnormal expression of CD4 T cell subsets in peripheral blood of patients with ovarian cancer and an imbalance of Th1/Th2 and Treg/Th17 provide a basis for clinical immunotherapy of ovarian cancer [33]. In addition, Su et al. identified lncRNAs and genes related to the prognosis of gastric cancer and showed that prognostic genes positively related to mortality were enriched in the CAM signaling pathway by performing a functional enrichment analysis [34]. Furthermore, Talia et al. showed that GPER levels are related to metastasis and premigration genes belonging to the CAM signaling pathway in breast cancer [35], which further suggested that T cell-related genes may be related to the progression of liver cancer via Th1- and Th2-cell differentiation and CAM signaling pathways.

*EOMES* is an important transcription factor that regulates the differentiation and function of effector T cells, and previous studies have shown that the survival of tumor patients is closely related to *EOMES* expression. For instance, Gao et al. suggested that the potential anticancer functions of *EOMES*, *ATF5*, and *ECM1* were confirmed by siRNA experiments [36]. The CD8 T cells were divided into three distinct subpopulations: PD1Hi, PD1Int, and PD1-. Ma et al. showed that compared with adjacent non-tumor liver tissues, the PD1Hi CD8 T cells were significantly enriched in tumors, and the PD1Hi CD8 T cells in liver cancer were highly expressed with depletion-related inhibitory receptors (TIM3, ctla-4, and so on) and transcription factors (EOMES, BATF, and so on) [37]. In the present study, *EOMES* was a T cell-related gene associated with liver cancer prognosis. Moreover, *EOMES* was regulated by has-miR-23b-3p and has-miR-23b-3p was regulated by lncRNA AC104820.2 in activated memory CD4 T cell-related genes ceRNA network, and *EOMES* was regulated by has-miR-23a-3p and has-miR-23a-3p was regulated by lncRNA AC000476.1 in CD8 T cell-related genes ceRNA network. Zaman et al. found an inhibitory effect of miR-23b-3p on the expression of the *PTEN* gene in renal carcinoma [38]. Meanwhile, Wen et al. showed that osthole inhibited EMT-mediated metastasis of prostate cancer by inhibiting snail signaling and miR-23a-3p [39]. Considering these results together, we speculate that *EOMES* may be the potential target of has-miR-23b-3p and has-miR-23a-3p in liver cancer and that they play important roles in the progression of the disease.

Moreover, in the present study, *CST7* was a T cell-related gene associated with liver cancer prognosis. *CST7*, also known as *CMAP*, has been reported to have close connections with liver cancer. For instance, Zhou et al. suggested that *CST7* and *CSTB* genes may serve as potential prognostic and diagnostic biomarkers for liver cancer [40]. Interestingly, *CMAP* is a novel cystatin-like gene involved in liver metastasis [41]. CD5L is a soluble scavenger cysteine-rich protein that regulates the inflammatory response. Aran et al. suggest that *CD5L* is upregulated in hepatocellular carcinoma and promotes the proliferation and anti-apoptotic response of liver cancer cells by binding to HSPA5 (GRP78) [42]. Thus, we speculate that *CST7* and *CD5L* contribute to liver cancer progression.

In the present study, immune-related prognostic indicators and chemical small-molecule**–**target interaction pairs associated with liver cancer were screened, which could guide liver cancer clinical decision-making. Moreover, we constructed a ceRNA network of immune-related genes associated with liver cancer prognosis and predicted miRNA targets, which provided a basis for the study of tumor-induced T cell-mediated immune escape. However, we did not use molecular biological experiments to verify our results. The molecular mechanisms that T cell-related genes influence the prognosis of liver cancer have not been investigated, and further molecular biological experiments are needed to explore the specific roles of these genes.

## Conclusion

T cell-related RNAs EOMES, CST7, CD5L, has-miR-23b-3p, and has-miR-23a-3p may be associated with the prognosis of liver cancer. And the molecular characteristics of these T cell-related genes were plotted by PPI network, ceRNA network, chemical small-molecule–target network.

## Supplementary Information

**Additional file 1: Supplementary file 1.**

**Additional file 2: Supplementary file 2.**

**Additional file 3: Supplementary file 3.**

**Additional file 4: Supplementary file 4.**

**Additional file 5: Supplementary file 5.**

## Data Availability

Not applicable.

## Abbreviations

TCGA: The Cancer Genome Atlas
KEGG: Kyoto Encyclopedia of Genes and Genomes
GO: Gene ontology
PPI: Protein-protein interaction
BP: Biological process
ESTIMATE: Estimation of STromal and Immune cells in MAlignant Tumor tissues using Expression data
*CD5L*: CD5 molecule like
*EOMES*: Eomesodermin
*CST7*: Glutathione S-transferase
*CD69*: CD69 molecule
*ZAP70*: Zeta chain of T cell receptor-associated protein kinase 70
